# On the Treatment of Cholera

**Published:** 1866-04

**Authors:** D. B. Trimble

**Affiliations:** Chicago


*On the Treatment of Cholera.* By D. B. Trimble, M. D., of Chicago.

As the subject of Cholera is now occupying the medical as well as the public mind, it may not be out of place for those in the profession who have had an opportunity of witnessing its ravages, to give their experience and opinions respecting its treatment. Having seen it to a greater or lesser extent in each of the four epidemics that have prevailed in the United States since 1832, I have been compelled to study it, both in the writings of others and by observation at the bedside. I therefore propose to give a brief relation of the treatment as practised by some prominent authorities, and also my own, hoping it may be of use to some of the numerous class of young physicians who recently left this city to enter upon the pursuit of our arduous profession.

Its semiology and treatment are what practically concerns the physician, and though it would be very gratifying to understand its etiology, it is not of essential importance. Whether it is contagious or non-contagious, or purely epidemic, is a matter of more interest, but from the conflicting opinions prevailing on the subject, cannot be definitely determined without more critical observation. My own opinion on this subject, is, that if it does not appear amongst us, it will not be owing to quarantine regulations however strict. Still, there can be but little doubt that proper sanitary regulations in cities will greatly abbreviate and lessen its ravages.

Dr. William Aitken, of Edinburgh, in his recent work on the "Science and Practice of Medicine," on the subject of epidemic influence and laws, says: "The second characteristic feature peculiar to some of the miasmatic order of zymotic diseases is, that they sometimes spread rapidly so as to incapacitate and destroy great numbers of people. The disease is then said to be epidemic. No subject has afforded greater scope for speculation, than the origin, cause and progress of epidemics. It is in vain to speculate upon the subject; and in the words of Dr. Wood, of Pennsylvania, 'all we can say with certainty regarding epidemics is, that there must be some distempered condition of the circumstances around us, some secret power that is operating injuriously upon our system, and to this we give the name of epidemic influence or constitution,' which is believed to predispose to the receptivity of the specific disease poison." "The most recent speculation regards the discovery of a peculiar atmospheric condition, ascribed to a principle called *ozone*, of which, as yet, we know nothing definite." In regard to the laws of epidemic influence, he says: "This influence frequently predisposes to disease, apparently independent of any other known cause, as in the case of influenza, or *cholera*." Sometimes the epidemic influence manifests itself by different types, and "may demand different and even opposite modes of treatment." During epidemics other diseases are apt to assume more or less of the prevailing epidemic features. Thus when cholera prevails, looseness of the bowels often complicate the course of other affections. "The first effects of the epidemic are usually the most violent and marked." "The epidemic influence sometimes disappears entirely after a short prevalence; sometimes continuing with irregular intervals for several years, as witness influenza and cholera." The cholera first appeared in the Eastern States in 1832; reappeared in 1834; then disappeared as an epidemic until 1849; returned again in 1854, and has not appeared since, except sporadically. In the above mentioned circumstances relative to the laws of epidemics, all observing practitioners must coincide; and also that "the lower animals are also subject to epidemic influences; and seasons of unusual fatality among them have coincided with those in which the human family have suffered." Witness the "cattle plague" now prevailing in Europe. A chapter in his work "on the management of epidemics," is well worthy of perusal by not physicians only, but by all who have control of municipal affairs, but it is too lengthy for quotation. The recommendations may be summed up in enforcing cleanliness, temperance, ventilation, the use of pure water, disinfectants and equanimity of mind. In regard to cholera, Dr. Aitken thinks it both epidemic and contagious. Dr. Flint, in his new work on the "Practice of Medicine," contends against its contagious nature. (Page 425.) Dr. Wood says: "The essential and specific cause of this" (Asiatic) "form of cholera, is undoubtedly an epidemic influence." "The nature of the *specific* cause is unknown." "The hypothesis that cholera is propagated by contagion has not been without numerous advocates." This hypothesis he endeavors to confute. His enumeration of the predisposing and exciting causes coincide so thoroughly with my own observation that I am induced to quote them. In relation to the first class, he remarks: "The *predisposing causes* are very numerous. Whatever is calculated to reduce the vital energies, or to reduce the vital actions below the standard of health, may be ranked among these causes. Not only are debilitated individuals in greater danger when laboring under this disease; they are also much more liable to its attack. Previous disease, old age, intemperance, vicious indulgences of all kinds calculated to impair the health, deficient alimentation, an exclusive vegetable diet, confined air, especially in low and damp places, the effluvia of crowded residences, continued grief, *fear*, anxiety and other depressing emotions, all predispose to cholera." "Nor are the *exciting causes* less numerous." "Whatever makes a sudden and powerful impression on the system or irritates the stomach;" "exposure to cold while our body is warm;" "indigestible food;" immoderate eating; "unwholesome drinks, as impure water or impure liquors of any kind;" "very cold drinks or ices, taken too freely;" "purgatives, especially drastic," etc. In the last epidemic that visited this country, I remember attending a portion of a family of nine persons, (part was attended by a homœpathist) five of whom in the course of a week were taken with cholera; three died after a few hours sickness,—one after four days, and one, a little girl of eight years of age, recovered. The last mentioned (who was my patient) was taken from the house before she was attacked. They were temperate people, and lived moderately well, though the house was small for the size of the family, and the yard very small, containing a well of water with a pump, and also a privy. Upon examination of the premises by the mayor, it was found that the liquids from the privy had percolated through the earth, and mixed with the water in the well. This terrible impurity, the slight ventilation of the building, the dampness of the situation, (being near the Delaware River,) and the epidemic influence that had just descended on that locality, were probably the causes of the great malignancy of the disease.

In support of my belief, that quarantine regulations are of little or no avail in preventing the presence of the cholera, I will quote the opinion of the Epidemiological Society of Great Britain in regard to it, and which may be found in the *Medical News and Library*, for November, 1865. It says: "With respect to any endeavor to exclude epidemic cholera by the system of quarantine, no reliance whatever can safely be placed upon it to keep off or prevent the pestilence. 'Sanitary precautions *within* a *place* are far more important than sanitary orders without.' "

Cholera is sometimes preceded for several days or hours, (as the case may be,) by a diarrhœa, but often comes on suddenly, with vomiting and purging; the former, and, after a few dejections, the latter, being composed of a clear liquid with white flakes floating in it, or milky appearance. There is sometimes nausea, though this is not an invariable accompaniment of the vomiting. Vertigo, tremors, great muscular debility and severe pains in the bowels succeed. There is great thirst and heat in the stomach. The urinary secretion is partially or entirely suppressed. The circulation is greatly depressed in the majority of cases, producing faintness, a feeble and rapid pulse, which often becomes imperceptible hours before life is extinct. There is depression also in the function of respiration, producing more or less dyspnœa, with a cool breath. The muscles are more or less affected with cramps, though there are cases in which this symptom does not occur. The surface of the body is cool and damp; sometimes there is profuse perspiration. The skin often assumes a cyanized or blue appearance; the features are contracted, and the extremities present a shrivelled appearance. The eyes are sunken and have a filmy appearance; the hearing is often obtuse; the voice very weak, or entirely suppressed; and the mind generally tranquil from its inertness. Sometimes reaction takes place, and there is considerable fever.

In regard to the treatment there is considerable difference of opinion among practitioners, though among standard authorities there is not so much. In regard to the prophylactic treatment, Dr. Flint says: "Attend to the diarrhœa; if treated early it can generally be checked; and if checked, the cholera does not generally appear." He uses anodynes, astringents, "rest and recumbancy." He recommends laudanum and spts. camphor; or tinc. opii camph. with tinc. kino, catechu, or krameria; or sulph. morph., with ac. tannic, or acet. plumb., bismuth and capsicum. I have used with very good success, in this stage of the disease, a combination of opium, camphor, capsicum and tannic acid, digested in the compound spirits of sulphuric ether.

In the first and second stages of the disease, or those preceding collapse, the indications for treatment are, according to Dr. Wood, "to arrest the evacuations from the stomach and bowels; to relieve irritation of the gastro-intestinal mucous membrane; to restore the suspended secretions, especially that of the liver; to equalize the circulation; to relieve the nervous disturbance; and to support, when necessary, the general strength. Of these, the most important is to arrest the alvine evacuations, for it is by their continuance and increase that the fatal condition of collapse is generally induced." To meet these indications it has been my practice to prescribe opium or some of its preparations, in connection with calomel or pil. hydrarg. (generally the former) in small and frequently repeated doses, and combined, if the case was urgent, with some astringent; as acet. plumb., kino, or krameria. In regard to the use of opiates, Wood, Eberle, Aitken, Flint, and a large majority of authors and practitioners, unite in approving. The three first mentioned authors, with many others, concur in the use of mercury. Dr. Flint does not recommend it; but I have seen too many cases, which, after the administration of calomel, and the return of the secretion of bile, and its appearance in the evacuations, have been gradually relieved from the diarrhœa and vomiting; from the cramps and pain; the temperature of the skin augmenting; the pulse improving, the strength increasing, and all the symptoms alleviated; not to attribute much of this improvement to the mercurial influence, and to believe, that without it, those cases would have been fatal.

In some cases, and those are generally the most fatal, there is little or no vomiting or diarrhœa, but the patients rapidly sink into the stage of collapse.

All these authorities also recommend astringents; and plumb. acet., kino, krameria and catechu are the especial astringents recommended. Tannic acid is with me also a favorite astringent in this disease. The acetate of lead acts as a sedative to the bowels, as well as an astringent; the others by their astringency only. To allay the intense thirst, small pieces of ice may be frequently swallowed, but large draughts of water only aggravate the vomiting. The ice will often alleviate the nausea, and for this purpose also, carbonic acid water, lime water, aromatic spirits of ammonia, or other aromatics, may be given; and a sinapism of full strength applied to the epigastrium. An enema composed of thin clear starch or linseed tea, with laudanum; with an astringent, if the case is very urgent, in addition, may be administered, with a view to checking the diarrhœa and alleviating the tormina. This may be repeated. To relieve the cramps, dry frictions may be applied, but stimulating frictions are objectionable, on account of the irritation of the skin soon becoming too great to bear them. If the feet and legs alone are cramped and cold, a warm stimulating foot-bath may be used. During the last epidemic, the thought occurred to me that anæsthetics might be applicable to this symptom, and I determined to use chloroform in the first favorable case for its administration that should present. About the last case I had that year, was, from the great severity of the cramps, also the most suitable for the experiment. The patient, who was a strong muscular man, about thirty-five years old, sent for me in the morning, but being from home, I did not see him until about 11 o'clock A. M. I found him vomiting and purging the peculiar choleraic fluid; with the other symptoms of the second stage, and cramped apparently in every muscle of his frame. The muscles of his limbs, abdomen, thorax and face were contracted, giving him a most frightful aspect, and requiring three or four strong men to keep him on the bed, during his paroxysms. Though a man of firmness, his screams could be heard in neighboring houses. He resided three miles from the city, and I sent a messenger for four ounces of chloroform. His skin was cool, and pulse scarcely perceptible. I gave him laudanum and brandy, which he at once ejected, and I awaited the arrival of the chloroform, which was handed me while he was in a severe paroxysm of the cramps. I immediately poured some on a handkerchief and held it near his nose, moving it from side to side of the bed, as he threw himself about, in his agony. After several minutes he suddenly threw himself on his back, perfectly motionless, and with staring and fixed gaze, seemed scarcely to breathe. But presently the lids relaxed, the features became less rigid, and he fell into a quiet slumber. I sat by him for some minutes while he thus slept, when he awoke cramped, and exclaiming "more! more chloroform." I administered it again with a more speedy result, and with longer effect, and was then obliged to leave him; giving strict directions for the use of the chloroform, and warning his attendants of its power. I returned at 4 o'clock P. M., and found that they had used it *all;* but he was greatly relieved of the cramps, and I now commenced the use of calomel and opium, which he partly retained. The vomiting and purging were of less frequent occurrence, the cramp returned only at long intervals, and then more slightly; and in about thirty-six hours from the first administering of the calomel, there was the appearance of bile in the stools, from which time his symptoms were greatly alleviated, and he rapidly convalesced; and in one week from the commencement of the attack, he was down stairs. I believed then, and still believe, that the chloroform saved his life, for though I do not suppose it would have cured him, yet it alleviated the intense suffering, which of itself would have probably produced fatal effects, and gave an opportunity to administer other remedies that would not have been retained but for its effect. In the *Medical and Surgical Reporter*, for October 5th, 1861, are several testimonials to the good effects of anæsthetics in cholera. Dr. Rowell, of San Francisco, gave it, "with a view to relieve pain only; morphine and opium in enormous doses, having proved unavailing, being rejected by the stomach as soon as swallowed." In his case, like my own, the vomiting and cramps ceased, and "the appropriate remedies were tolerated by the stomach, until their therapeutic action was fully established, and the disease controlled." Dr. Thos. D. Mitchell, in his work on "Materia Medica and Therapeutics," relates the case of a physician who was seized with cholera, and who, having no other remedy at hand, inhaled it, went to sleep, and awoke in "a safe condition." Steady and firm extension of the muscles is another means of relieving cramps.

In extreme prostration the diffusable stimulants may be used, but with caution. The intemperate require them more than the temperate. The ethereal preparations are perhaps the best; but tinc. camphor, arom. spts. ammon., brandy or port wine, may be used with this view.

Leeching is recommended in some cases, both by Prof. Wood and Dr. Aitken. The former advises it in the earlier stages to the epigastrium, where there is tenderness or burning pain in the stomach; the latter, to the temples, when there is headache. In regard to bleeding by venesection, the general impression is against it, but some eminent physicians have strongly advocated it. I have an indelible recollection of the first case that I ever attended, (in 1832,) and that not as a physician, (for I had not then entered on my medical studies,) but as an interested nurse, for it was in the case of my father. He was a man of delicate constitution, and was just convalescing from a slight attack of fever, when he was attacked with cholera, the cramps attacking first the muscles of the throat, and gradually extending to the limbs, but not so severely cramped as many. Our family physician, who had attended him up to this time, was himself taken sick, and sent a younger gentleman. My father was rapidly sinking, when the doctor alarmed my mother by proposing to bleed him. This she objected to, knowing his peculiar susceptibility to the effects of bleeding, usually the loss of three or four ounces of blood causing him to faint. He requested a consultation, when an old medical friend of my father was called in, but he too objected to bleeding. After considerable argument, however, he was induced to give his assent. By this time the surface was cool and shrivelled, and the pulse at the wrist almost imperceptible. He was raised up in bed, and with intense anxiety I watched the process. At first the blood would scarcely flow, but gradually it increased, and as they sat on either side of him with their fingers on his wrists, I could see the doctors exchange gratified glances with each other, and after taking, what I then thought, an enormous quantity, (about 12 or 14 oz.,) my father was laid down greatly relieved, and his pulse restored to a moderate volume. He was afterwards cupped over the abdomen, and eventually recovered. This gentleman afterwards informed me, that the two first cases he treated, (a father and son,) both died. He gave them stimulants, opiates, etc. In subsequent cases he almost invariably bled, and his success was very great. In my own practice it has been seldom that I have resorted to it, and then in cases that did not enable me to decide for or against its propriety. Dr. Wood says: "In those rare cases in which the pulse is full and strong, especially if connected with convulsive symptoms, blood should be taken from the arm." But in my father's case the pulse was greatly depressed, and this was his physician's reason for bleeding; I suppose on the theory of internal congestion. Dr. George Johnson, Prof. of Medicine in King's College, in "Notes on the Pathology and Treatment of Cholera," takes the position that the collapse of cholera is not due to a drain of liquid from the blood, and strongly advocates free venesection as the remedy chiefly to be relied on. His arguments are very ingenious, and well worthy of perusal, but too lengthy for quotation. In regard to the effect of venesection on the symptoms of collapse, he says: "But what has been the effect of venesection in not one or two, but in a large number of cases of cholera, and in the hands of many practitioners?" "It is in the writings of the Indian practitioners, that the largest amount of evidence is to be obtained as to the influence of blood-letting in cholera. Scott makes the following remarks on this subject: 'The abstraction of blood, unless as an anti-spasmodic, is a remedy so little indicated by the usual symptoms of cholera, that its employment in the cure of this fatal disease has afforded a signal triumph to the medical art.' It requires no common effort of reasoning to arrive at the conclusion that, when the powers of life appear to be depressed to the lowest degree, the pulsation of the heart all but extinct, the natural heat of the body gone, and the functions of the system suspended and incapable of being revived by the strongest stimulants, the abstraction of blood might yet prove a remedy against a train of symptoms so desperate." "Few remedies, on fair trial, have been more generally or unequivocally advocated than free blood-letting; and the most that has been urged against it is, that it is not always successful." He then quotes reports from medical officers, furnishing very striking testimony as to the benefit of bleeding in cases of extreme collapse. Annesley says: "In place of syncope being produced by bleeding, in the cases which I have treated, the pulse has invariably improved, and the feelings of faintness and debility disappeared." Bell makes the following statement: "The effect of blood-letting would indeed sometimes appear almost miraculous. A patient will be brought in on a cot, unable to move a limb, and, but that he can speak and breathe, having the character both to touch and sight, of a corpse; yet will he, by free venesection alone, be rendered in the course of half an hour, able to walk home with his friends." Rogers gives the following description of the effects of venesection by a medical man, who was himself the patient: "There was a sensation which I am at a loss to describe, as if my heart was ceasing to beat, and a dread of suffocation; this sensation was instantly relieved by bleeding, and I recovered immediately." After giving still farther testimony to this treatment, he says: "I maintain that the numerous well authenticated instances of great, and immediate, and permanent relief by means of venesection in the collapse of cholera, are irreconcilable with the hypothesis in question, viz: the loss of the liquid constituents of the blood." Dr Johnson's article is reported in the *Medical News and Library*, for January, February and March, 1866.

*Emetics* are never proper except in the forming stages, when, if the stomach has been filled with undigested food, a gentle emetic,—of infusion of mustard, or of ipecacuanha, may be used. In regard to *purgatives*, there is a difference of opinion respecting their use. Dr. Wood limits their employment to the mildest laxatives, if costiveness should be produced by the means used for checking the disease. Dr. Johnson, in the article above alluded to, advocates giving both cathartics and emetics, contending that the fatality is less than where opiates and brandy are employed. He chiefly uses castor oil as a purgative; Dr. Wood, rhubarb. In the stage of collapse, active stimulation, judging by the symptoms alone, would seem to be called for, but experience has shown that it cannot be used largely. In the opinion of Dr. Johnson it is positively injurious, but Drs. Flint and Wood advise it moderately; and the two latter physicians, as well as Dr. Aitken, recommend in this stage as external applications, warm blankets, hot bottles, sinapisms, astringent applications to check the profuse perspiration, etc. Bicarbonate of soda, weak animal broth with salt, injections of salt water and laudanum ; to allay vomiting, chloroform, ether, creosote, etc. ; to promote reaction, the essential oils in alcohol, diffusable stimuli, etc.

When *reaction* is established, "the treatment must be made to conform to the variable morbid conditions presented, and must be guided by the general principles applicable to other affections." (Wood.) Much care in the diet should be observed until the patient recovers fully his wonted health. As a prophylactic, Dr. Salisbury, of Illinois, recommends cider, and other diuretics. ("*Journal*," for November, 1865.)

A peculiar and, as claimed, a remarkably successful mode of treatment has been practised by Dr. John Chapman, of Southampton, England. It consists chiefly in the application to the spine of bags of ice. Occasionally he alternates with *warm* water. He relates seven cases, five of which were successful. No doubt this measure would have a powerful influence, whether for good or for evil my experience will not enable me to say. During the epidemic of 1849, I was called to attend on an estimable young physician, residing about three miles from my home, but being absent on professional duty did not see him until nearly six hours after the first symptoms. He was then collapsed and speechless, though not insensible, and had directed his own treatment ; a portion of which was the application to his abdomen of cloths saturated with ice water. He died about two hours after I first visited him.

